# Investigating Children’s Ability to Express Internal States through Narratives and Drawings: Two Longitudinal Studies during Pandemic

**DOI:** 10.3390/children9081165

**Published:** 2022-08-03

**Authors:** Giulia Vettori, Costanza Ruffini, Martina Andreini, Ginevra Megli, Emilia Fabbri, Irene Labate, Sara Bianchi, Chiara Pecini

**Affiliations:** Department of Education, Languages, Intercultures, Literatures and Psychology, University of Florence, 12 Via di San Salvi, Building 26 (Psychology Section), 50135 Florence, Italy; costanza.ruffini@unifi.it (C.R.); martina.andreini@stud.unifi.it (M.A.); ginevra.megli@stud.unifi.it (G.M.); emilia.fabbri@stud.unifi.it (E.F.); irene.labate@stud.unifi.it (I.L.); sara.bianchi2@stud.unifi.it (S.B.); chiara.pecini@unifi.it (C.P.)

**Keywords:** narratives, drawing, psychological lexicon, emotions, primary school children, pandemic

## Abstract

The COVID-19 pandemic emergency has challenged children’s socio-affective and cognitive development. It is essential to capture the modulation of their emotional experience through ecological and children-friendly tasks, such as written narratives and drawings. This contribution investigates the impact of pandemic experience (2020–2021 waves) on the internal states and emotions of the primary school age children, according to a longitudinal research approach through narratives (study 1 *n* = 21) and drawing tasks (study 2 *n* = 117). 138 Italian children were examined during COVID-19 three (study 1) or two waves (study 2). Children’s written narratives were codified on the basis of narrative competence and psychological lexicon. Children’s drawings were codified based on social/emotional, physical, and environmental elements. Results of narrative texts showed a lower psychological lexicon relating to positive emotions and a greater psychological lexicon relating to negative emotions only in the study sample group during the first lockdown compared to the previous and subsequent periods. Children’s drawings of themselves showed a decrease of negative emotions during the third pandemic wave in comparison to the first pandemic wave. Results inform mental health services, school practitioners, and parents about the importance of written narratives and drawings for promoting well-being in the developmental age.

## 1. Introduction

The COVID-19 pandemic, caused by the SARS-CoV-2 virus, has abruptly changed the daily life of the global population, which has experienced repeated periods of social isolation with a sudden limitation of personal freedom and changes in the main dynamics of relationships. These difficult conditions have made the present historical period particularly critical for socio-emotional functioning, and especially for children, who are engaged in multifaceted developmental dynamics [1]. The lives of children and young people have been disrupted in many ways, as efforts to contain the pandemic have required the closure of many developmental contexts, such as schools, childcare centres, colleges, recreation centres, libraries, sport centres, and entertainment centres. The isolation and restricted environments negatively impacted on children’s play [2], friendship relations, and social connectivity [3]. Clearly, the school closures significantly contributed to stress in children and parents [4] challenging children’s growth and development. According to the Center for the Study of Traumatic Stress [5], during the first quarantine period (March–June 2020), many children activated regressive behaviours and showed states of anxiety, anger or agitation due to the loss of reference routines. Anxiety and frequent alterations in sleep-wake rhythms, nightmares [6], repeated enuresis, mood changes, including excessive crying or episodes of excessive anger, fear, or sadness, as well as a progressive decrease in interest in activities that, before the emergency period, were considered by the children themselves as pleasant, were recorded [5].

There is a clear need for more research that evaluates children’s emotional perspective during stressful events such as pandemic outbreak [7]. In order to identify children’s intense distress experience, monitor, and prevent such experiences from perpetuating and having a clinically relevant evolution [8], it is necessary to adopt actions which might enhance the activation and reinforcement of resilience in children’s adaptation to stressful life events at an early stage, on an individual and collective scale.

To explore children’s internal states during the pandemic, narratives [9] and drawings [10] are useful, ecological, and unobtrusive tasks, commonly linked to the school context, capable of giving children a voice, accessible even to the youngest children and practicable even in online education [11]. Narratives and drawings can be potentially sensitive to the emotions that the child has experienced following the closure of school and educational environments and prolonged home confinement in the family context. Conversely other instruments, such as direct observation scales, questionnaires, or interviews, that require conscious access to emotions and their explicit verbalization, may be not sufficiently sensible or not appropriate for young children.

### 1.1. Psychological Lexicon in Children’s Narratives

Narrative writing is seen as a source of data for understanding children’s internal states and emotions, in addition to cognitive-linguistic aspects. It is a common practice involving preschoolers and school-aged children to create an invented story [12]. This provides the possibility to examine large scale samples of children since the early years of life. There is a large amount of research-based evidence showing that narratives are an effective means to bring out emotional experiences [13,14,15] that a child would not be able to express as a result of direct questions. Narratives are made up of fragments of the writer’s self-expression through projective identification, structured and embodied in fictional characters, landscapes, ideas, concepts, and emotions [16]. The construct of psychological lexicon has proved useful in studying and analysing the emotions prevalent in children’s narratives [17] and it refers to a particular form of language characterised by nouns, verbs and adjectives not referring to real objects, but to own and others’ internal states [18]. For example, the use of words such as “happy” (positive emotion), “hope” (willingness state), and “becoming friends” (socio-relation) refers to internal states reflecting multiple domains. The ability to refer to internal states evolves from the earliest stages of linguistic development and is subsequently consolidated in relation to the process of schooling and activities such as composing and understanding texts [13,19]. During preschool years and middle childhood, cognitive development (e.g., working memory and executive functions) enhance children’s theory of mind understanding and use of psychological lexicon [20]. Around the age of 5 years, the child reaches the ability to refer to the psychological reactions of characters in narrated stories. Its presence and development in oral [21] and written [22] narratives are widely documented in preschoolers [19,23] and school-aged children [24]. It is from this age onwards that children tend to recall more frequently the wishes, intentions, aims and emotions of the protagonists described in the stories. During the last year of primary school, in particular, the cognitive psychological lexicon becomes richer [24] and more varied as children use more complex terms with metacognitive connotations (e.g., reflecting, devising, being puzzled) [25,26]. This tendency progressively consolidates over the course of school age; a clear indication of an increasingly sophisticated ability to use language [27]. During school age, moreover, there is a significant decrease in the use of psychological lexicon relating to perceptual and volitional states and a substantial increase in cognitive ones. The psychological lexicon the child uses in inventing a story is also strongly influenced by the use of oral or written language [28], by the motivation to write, the climate and the situations in which one narrates [26,29]. Considering children’s ability to express internal states within their narratives [25,26], it is of paramount importance to implement this task to investigate children’s internal states linked to COVID-19 pandemics. Children can be involved in inventing narrative text to stimulate the access to their personal experience by inventing a story without precise constraints that could limit their reflection on their own experiences and emotions. Invented stories represent a narrative context particularly favorable to stimulate and promote children’s emotional involvement and identification with story characters’ emotions and feelings [30]. Essentially, the story represents a direct expression of the psychological experience of the writer [31], and means through which children can express, more or less consciously, their emotional states [25,32], using terms that imply links with their theory of mind [33].

### 1.2. Drawing Oneself: Children’s Representations of Themselves in Challenging Life Circumstances

Drawing is a privilege tool to capture children’s representations about personal experiences and feelings [34], and a means of communication of children’s knowledge about the social world [10] which is moved by thoughts, emotions, and feelings [35]. Drawing serves as an important means of communication to children about their ideas on relationships [36], specific circumstances (e.g., conflict situations [37]), and self-image at different times (e.g., the subject in the present vs. the subject in the past [38]). The drawing also represents a practice that might enhance children’s emotion regulation strategy, undertaken by the subject as a way of self-management in moments of strong emotional activation [39].

Children engage in drawing from a very early age, and their drawing abilities develop quite early [36]. During middle childhood, children are able to draw familiar objects and moreover their capacity to enrich and modify drawings in a personal way improves [40]. A large number of studies have examined the development of children’s ability to represent themselves in different circumstances (relationships with friends, e.g., [41]; school, e.g., [42]) systematically examining whether and how drawings show some properties, such as the characteristics of the figures (e.g., sex, similarity), the characteristics of the relationships (e.g., cohesion, emotions, conflict), and the context (e.g., the environment). A few studies, however, provided data about children’s drawings during the COVID-19 pandemic to investigate social, emotional, and contextual properties of drawings [43]. As an exploratory step in this new direction, in this study, children’s drawings about themselves were analyzed in terms of use and presence of social/emotional, physical, and environmental elements in primary school children’s drawings. *Social and emotional* elements are shown in the drawings by the presence of one or more other figures as well as by their spatial proximity as an index of the figure’s commitment. In this respect, we wondered if the use of different colors in children’s drawing might add useful additional information in relation to the emotional area [44]. By *contextual* elements we mean the examination of the setting in which the child represents him/herself (e.g., internal vs. open space) and the number of pandemic tools (e.g., face mask, hand disinfectant) in the drawings.

As a privileged tool of self-expression and communication, children’s drawings can contribute to our understanding of children’s representation about themselves in different conditions, for example when living in impacting life circumstances, such as a pandemic, in terms of individual and contextual features (e.g., social, emotional, and contextual aspects in drawings).

### 1.3. Rationale

The isolation and restricted environments imposed by COVID-19 pandemic have produced significant changes in children’s socio-emotional life. Yet, there has been little research examining children’s internal states and emotions coinciding with the pandemic outbreak by using ecological and children-friendly tasks. Thus, the present longitudinal research was aimed at verifying the impact of pandemic experience on the internal states of the primary school age children according to a longitudinal research approach through narratives (study 1) and drawing (study 2) tasks. In detail, this general objective is pursued through two longitudinal and independent studies as follows:

1. The first study was aimed at investigating the existence of both quantitative (i.e., the number of terms) and qualitative (i.e., the variety of terms referred to mental states of different domains, e.g., emotion, volition) changes in psychological lexicon in children’s narratives along three-time points during the pandemic (before, during, and after the first lockdown);
2. The second study was aimed at analysing changes in internal states in children’s drawings along two-time points during the pandemic (the first pandemic wave and the third pandemic wave).

This study is part of a larger project aimed at assessing the emotional states of children through drawings and narratives; following the COVID pandemic, the conditions have led to the collection of more specific data in response to the pandemic situation. Thus, the objective of the present study to use narratives and drawing to evaluate the emotional states expressed by children during the pandemic situation was defined after the onset of the pandemic emergency, following qualitative observations on the sudden changes expressed by children in their productions.

The study was conducted according to the guidelines of the Declaration of Helsinki, and was approved by the University of Florence ethics commission and school Directors. Parents of the children and children themselves provided their consent to participate in the research.

Study 1. Children’s Narratives during the Pandemic.

## 2. Materials and Methods

### 2.1. Participants

From the total sample of 138 school-age children, a subgroup of 21 children (study sample group [SSG]; 7 F, 14 M, mean age 6.85 years, SD = 37) performed the narrative task three times during the pandemic.

**Figure 1:**
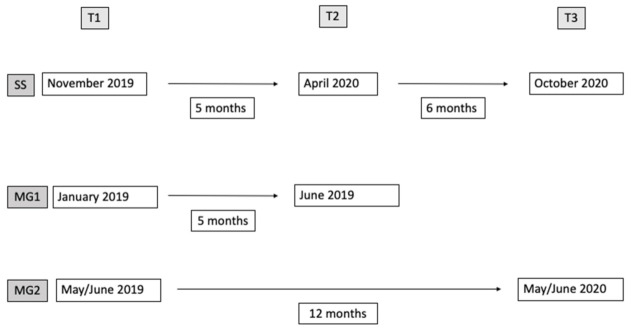
Study design.

Data collection started when children were attending grade II of a primary school in Florence, Italy. The class involved in the study was selected thanks to a collaborative relationship between the researchers and the class teacher. They were followed longitudinally for five months between the first (T1, November 2019) and the second assessment (T2, first lockdown in April 2020) and for the subsequent six months between the second (T2, April 2020) and the third assessment (T3, October 2020) when the children had moved to grade III. Moreover, data from two groups of children (matched group 1 [MG1] and matched group 2 [MG2]) were used as a control condition to compare the EG changes in narratives during pandemic to the MG changes in narratives during a non-emergency situation. The two groups were selected from a larger sample who performed the narrative test before the pandemic emergency according to a longitudinal design at five (MG1) and twelve months (MG2) intervals (see Figure 1). Children in the two groups were selected retrospectively on the basis of the class attended and narrative skills in order to make both groups comparable to the study sample group. Specifically, children in MG1 (14 F, 6 M; mean age 7.24) were assessed in grade II of primary school in January 2019 (T1) and then, in June 2019 (T2), five months apart. Children in MG2 (7 M, 14 F; mean age 7.04) were assessed in grade II of primary school in May/June and then, approximately one year later, at the end of grade III of primary school (May/June).

In all groups, children were Italian native speakers, and none had developmental disorders or learning disabilities.

### 2.2. Description of the Selected Tasks

#### 2.2.1. A Parent Report Questionnaire

Parents of the children in the study sample group were asked to fill an online questionnaire aimed at describing significant emotional and behavioral aspects of children’s daily life during the first lockdown due to COVID-19 outbreak (April 2020). Information collected is useful to outline children’s profile of resilience and to gain an overview of the adaptive resources that children show from their parents’ perspective. The structure of the questionnaire was adapted from the study by Pisano and Cerniglia [45] aimed at analysing the impact, in the opinion of parents, of COVID-19 restrictive measures on the lives of their school-aged children. The questionnaire is divided into three areas, each consisting of four questions, for a total of 12 questions. The first area investigates children’s *regressive behaviours* with particular reference to the loss of some previously acquired skills (e.g., the habit of sleeping alone in one’s room, sphincter control, language adequacy or the manifestation of fears). The second area explores the child’s *protest behaviours* in relation to the sudden change in lifestyle (e.g., irritability, frequent mood changes, sleep disturbances, low tolerance to restrictions and information about the pandemic). The third area investigates children’s *adaptive responses* regarding states of calm, tranquility, balance and adaptation to restrictions.

#### 2.2.2. Written Narrative Task to Assess Psychological Lexicon

Children were asked to write an invented story [15] during the school time. Any time or text length restrictions were provided. The children wrote down on a sheet of paper taken from their exercise book and were free to revise the text or copy the text draft into a final version. No instructions, suggestions or help were given on the narrative story plot to be tackled and the children were instructed to express themselves in a free way and that their narratives were exempt from assessment.

Regarding children in the study sample group, the first assessment was carried out in the school environment, during normal classroom teaching activities (T1), the second assessment was administered in distance learning mode and the texts were subsequently sent by the parents to the teacher via email (T2-first lockdown); finally, the third assessment was carried out during classroom teaching organised according to the regulations in force at the time of the second pandemic wave (T3).

Regarding children in the MG1 and MG2, they wrote their narratives in the classroom during the standard teaching activity at time intervals corresponding to T1-T2 and T1-T3, respectively.

The psychological lexicon in children’s narratives was coded basing on the 8-categories decoding system ([26,46]; see Appendix A for a more detailed description) as follows:

- physiological states (e.g., being hungry, being born)
- perceptual states (e.g., seeing, hearing)
- positive emotional states (e.g., happy, content)
- negative emotional states (e.g., angry, sad)
- volitional states (e.g., wanting, desiring)
- moral states (e.g., good, duty)
- socio-relational states (e.g., joking, helping)
- cognitive states (e.g., thinking, knowing).

The number of terms referred to psychological lexicon including repetitions (e.g., “sad”, “happy”, and “to wish” result in a score of “3”), as well as the variety of terms referred to mental states of different domains (e.g., “sad”, “happy”, and “to wish” result in a score of “2”) were recorded.

Moreover, children’s narrative competence was checked. For each written narrative, the following measures of narrative skills were used ([15]; See Appendix B for narrative examples produced by children):

- Text length in number of words.
- Narrative structure defined on the basis of eight constituent elements: story title, conventionality of the opening, character definition, setting definition, problem definition, unfolding, problem solution, conventionality of the conclusion. On the basis of the presence, absence or combination of the above elements it is possible to identify five levels of increasing degree of structural complexity: no writing (score 0); non-story (score 1); sketch story (score 2); incomplete story (score 3); essential story (score 4); complete story (score 5). Two independent judges coded the material. Interrater agreement was 90%.
- Cohesion level given by the number of temporal (e.g., once upon a time, when, never, before, never again, from that day, in a moment, always, after) and causal (e.g., thus, because, so) connectives, balanced for the total number of words. Two independent judges coded the material. Interrater agreement was 99%.
- Coherence level given by the calculation of inconsistencies between one clause and another, balanced for the total number of propositions. Two independent judges coded the material. Interrater agreement was 95%.

### 2.3. Data Analysis

The results of the questionnaire [45] were qualitatively inspected to describe the principal SSG children’s behavioural changes reported by parents at the first lockdown. The responses were analyzed at the group level.

Given the size and age of the sample, non-parametric statistics were planned for the analysis of psychological lexicon and narrative skills. Considering that young primary school children’s narrative skills are emergent, a non-normal distribution of the collected measurements is expected.

According to the aim of this study 1, the non-parametric Wilcoxon test for paired groups was used separately in the three groups (SSG, MG1, MG2) to test for significant differences in the use of psychological lexicon at the different assessment time points.

Analyses were conducted with the software SPSS v. 26.1.

### 2.4. Results of the Study 1

All participants in the sample completed the task, therefore, no missing data were recorded.

After the qualitative inspection of the data collected by the questionnaire in the study sample group, none of the children’s difficulties in adapting to the lockdown restrictions were observed by parents. For example, at item 7 *“Has your son/daughter manifested fears in the last month that he/she did not have before?”* (Area 1-regressive behaviors) 52.4% of parents responded “never” (23.8% “rarely” and 23.8% “occasionally”); at item 7 *“Has your son/daughter experienced any sleep problems in the last month that you had not observed before (difficulty falling asleep, restlessness during sleep, frequent awakenings)?”* (Area 2-protest behaviors) 57,1% of parents responded “never” (28.6% “rarely” and 14.3% “occasionally”); at item 11 *“In the last month, has your son/daughter seemed able to adapt to the restrictions brought about by the pandemic?”* (Area 3-adaptive responses) 61,9% of parents responded “often” (19% “occasionally”, 14.3% “always”, less than 5% “rarely”).

Previously to the examination of the psychological lexicon in children’s narratives, narratives skills (structure, cohesion, and coherence) were checked to ensure the comparability between study sample and matched groups. The descriptive data of the narrative skills obtained in the children of the study sample group across pre-, during-, and post- first lockdown are presented in Table 1.

The results of the qualitative inspection of the data suggests that the narrative skills were comparable between the matched-study sample groups, in line with results from previous studies on second graders. In agreement, the composite score reported by Gamannossi and Pinto [25] falls within the Confidence Interval (95%) of the composite score obtained by SSG at T1 and T2, respectively. Similarly, the difference in the composite score obtained on average by SSG in one year (T3-T1 = 86) was equal to the mean value of change between one school grade and the next one as described by Gamannossi and Pinto [25].

Regarding the results about the use of psychological lexicon by children in their narratives, the descriptive data of the psychological lexicon measured in the SSG at the three observation times (pre-, during-, and post- first lockdown), and the results of the analysis with Wilcoxon’s W-statistic are presented in Table 2.

The results show a statistically significant decrease in the number of terms referred to positive emotions used by children in their narratives and a significant increase in the number of terms referred to negative emotions used by children in their narratives to refer to fictional characters’ internal states between the pre-lockdown (T1) and lockdown (T2) periods. From a qualitative inspection it emerges that terms referred to positive states changed on the whole groups from 38 positive states at T1 to 16 at T2 (T1: 4 have fun, 11 happy, 10 nice, 7 happy, 1 courage, 1 fall in love, 1 celebrate, 1 wonderful, 1 kiss; T2: 3 having fun, 1 kissing, 7 happy, 2 nice, 1 content, 1 beautiful, 1 calm), and terms referred to negative states showed the opposite patterns from 6 at T1 to 19 at T2 (T1: 2 sad, 1 angry, 1 crying, 1 fearful, 1 frightening; T2: 2 being afraid, 4 bored, 2 frightened, 3 crying, 3 sad, 2 angry, 1 worrying, 1 ugly, 1 complaining).

Apart from the category of emotions, no other category of the psychological lexicon registered significant differences between the pre-lockdown (T1) and lockdown (T2) periods.

No significant differences emerged from the comparison between post- (T3) and pre- (T1) lockdown periods, whereas a significant decrease in the use of terms referred to negative emotions and cognitive states used by children in their narratives to refer to fictional characters’ internal states resulted from the comparison between the post-lockdown (T3) and lockdown (T2) periods.

The changes in narrative skills (number of words and structure) for MG1 and MG2 groups, at the respective times of detection are reported in Table 3.

Table 4 shows the descriptive data of the psychological lexicon detected in the MG1 and MG2 groups at a distance of about 5 (MGT1-MGT2) and 12 months (MG2T1-MG2T2), respectively. Comparison of the psychological lexicon recorded at the two measurement times suggests that there were no significant differences in the use of terms referred to emotional states by children in their narratives in either group, MG1 or MG2.

Study 2. Children’s Drawing during Pandemic.

## 3. Materials and Methods

### 3.1. Participants

From the total sample of 138 school-age children, it was selected a sub-group of 117 children (67 F and 50 M; average age 8.55 years, DS = 1.01; 114 with typical development, 1 with developmental coordination disorder, 1 with dyslexia, and 1 with hyperactivity/attention deficit disorder). Children attended primary schools in Tuscany region, Italy; 31 children attended second grade, 20 children attended third grade and 66 children attended fourth grade. Three classes were selected to have a representative sample of the primary school population as the study did not mean to investigate whether there were age differences linked to the pandemic situation.

This sample was tested once in the period of the third pandemic wave (January 2021–May 2021).

### 3.2. Description of the Selected Tasks

#### Drawing Task

Each child was asked to make two drawings on two white sheets with these instructions (adapted from [37]): “Represent yourself a year ago” and “Represent yourself today”. No further instructions, suggestions or feedback were given neither before nor during the task. The tasks, administered collectively and inserted within the daily teaching activity, were carried out without time limits and within school settings during normal face-to-face teaching activities. The two instructions were chosen in order to evaluate whether the social/emotional, physical, and environmental elements in drawings reflected variations in the memory of the first lockdown period with respect to the representation of oneself during the current period characterized by restrictions but absence of lockdown.

An expert and prepared rater adopted the following categorization by using a 6-point Likert scale (1 = absence; 6 =high presence) as follows:

*Social/emotional* aspects comprises two subscales each providing a continuous score (from 0= absence of the characteristic to 6 = high presence of the characteristic). The first subscale refers to the number of figure/s drawn; the second subscale refers to the distance the child draws between the child him/herself and the other figure/s, if present.

- *Presence of children*: Does the child draw him/herself alone or with other persons?
- *Proximity with other children*: Are the figures separated by a significant space?
  *Figure’s Color*: the use of colors was considered as a qualitative aspect in the figurative products of children. According to the coding system followed [46,47], black-gray colors and yellow-green colors were considered indicators of positive and negative emotions, respectively. From the scoring derived the index of “dark color” as the mean of gray and black scores and the index of “light colors” as the mean of yellow and green scores.

*Contextual* aspects comprise two subscales each providing a continuous score (from 0 = absence of the characteristic to 6 = high presence of the characteristic). The first subscale refers to the examination of the setting the child draws in his/her drawings. The second subscale refers to the examination of the presence of the face mask and of other pandemic tools (e.g., hand disinfectant).

- *Physical close environment*: Is the setting in which the child represents him/herself a close space?
- *Pandemic restrictions elements*: How many pandemic tools are represented by the child in the drawing (e.g., face mask, hand disinfectant)?

On the drawings of 30 children (34.5% of the whole sample) a double coding was completed by two independent raters. The disagreement was resolved through the discussion.

Two independent judges coded the material; interrater agreement was 100% after discussion.

### 3.3. Data Analysis

A non-parametric Wilcoxon test was used to investigate the existence of differences between the drawing properties examined in the two drawing tasks (i.e, “Represent yourself a year ago” and “Represent yourself today”) performed by the same children. Correlations between the Delta values (changes in the drawings between “Represent yourself today” and “Represent yourself a year ago”) of all the variables were studied with the non-parametric Spearman test.

Analyses were conducted with the software SPSS v.26.

### 3.4. Results of the Study 2

Mean and standard deviations of the collected measures are reported in Table 5. For 18 children, it was not possible to analyze the presence of closed space, because they represented only their face, without context.

The results of the non-parametric Wilcoxon test showed that in comparison to the drawing “Represent yourself a year ago”, in those “Represent yourself today”, the use of black and dark colors was lower. Also the gray color tended to reduce, as it was less represented in the drawing “Represent yourself today”, but without reaching statistical significance. In addition, for what concerns the contextual properties, there was a significant reduction in close environment and a higher presence of the pandemic restriction elements in the drawing “Represent yourself today” than in the drawing “Represent yourself a year ago”. No differences between the two time-point representations were shown for social/emotional properties.

From the analysis of correlations with Spearman Rho (Table 6) emerged that the Delta values (difference between “Represent yourself today” and “Represent yourself a year ago”) of the social/emotional properties positively correlated with light colors. For what concerns the contextual properties, delta of the presence of a physical close environment were positively related to dark colors and negatively related to light colors while delta of pandemic restriction elements did not correlate with any measures.

## 4. Discussion

The current longitudinal research investigates the impact of the pandemic experience on the internal states of the primary school age children according to narratives and drawing. In particular, a closer look at children’s written narratives (Study 1) and drawings (Study 2) showed a dynamic modulation of their emotional experience in the course of the pandemic, according to the change in contextual characteristics (e.g., distance learning in primary school) of pandemic life circumstance.

The first study was aimed at investigating differences in the use of psychological lexicon in children’s narratives among pre-, during-, and post- first lockdown. In the face of a normal developmental trajectory of narrative skills, the first result suggests that the quantity of psychological lexicon used in narratives remains rather stable over the time period considered, although a tendency towards an increase in the use of psychological lexicon was observed during the first pandemic period. Beside the analysis of the quantity of psychological lexicon in narratives, the quality in the use of terms related to psychological lexicon was also considered. Results about the presence of changes in the variety of psychological lexicon offer an interesting and more articulated picture. Specifically, during the period of the first lockdown (T2) the psychological lexicon used in the narratives underwent a significant change within the specific affective-emotional domain. Thus, a decrease in positive emotions and a parallel increase in negative emotions (e.g., sad, bored, angry) were found in the narratives written by children during the first lockdown (T2), coinciding with distance learning in primary school, compared to narratives written in the pre-lockdown period (T1). These emerging changes in the use of psychological lexicon interested the specific domain of emotions, as can be observed from the overall general stability of psychological lexicon in the other domains (e.g., volition, morality, social-relation). The decrease in the number of terms referring to positive emotions and the parallel increase in the number of terms referring to feelings of discomfort and uneasiness suggests that the children were able to recognise emotional experiences of a different nature (wellbeing vs. discomfort), just as they were able to recognise a change that had taken place in their emotional world, since the wellbeing of the fictional characters in their stories had decreased. This internal emotional change in fictional characters might be linked to the decrease in the possibility for the children themselves to experience positive emotional occasions in real life due to the dramatic situation of the pandemic [48]. Unexpected and adverse events till traumatic one’s “shake up” the mentalization and regulation of the emotional world [49] in such a way that the most difficult emotions to “think about” are positive emotions, while negative emotions are more easily accessible at a cognitive level. Past literature has outlined the dual function of negative emotions of anticipating and predicting future traumatic events, according to a mechanism that can be partially compared to that of hypervigilance, as well as of reflecting and organising one’s emotional experiences in a way that is recognised by the individual as having meaning and significance because it is closely connected to his or her life dimension [50]. A further relevant result is given by the measurements occurred in the post- first lockdown (T3), a period characterised by the continuation of the emergency, because of the second pandemic wave, but also by the return of face-to-face learning in primary school, for which the average number of terms of positive and negative emotional states was closer to those recorded in the pre- first lockdown period (T1), with the absence of significant differences between the two measurement times (T1 and T3). A possible line of interpretation of this result could lead to consider it as indicative of a change that is stabilised following the achievement of a new set-up that allows the child to adapt in a resilient way to the critical life situation she/he is facing in reality [3]. The reduction in negative emotional states at the end of the first lockdown delineates the lockdown as an unexpected adverse but non-traumatic experience because at its end the normal emotional setting was restored. This result is comfortable: the children were able to restore the full range of emotional resources once the source of their emotional distress was removed. The possibility to resume the path of emotional development can certainly be linked to different factors such as children’s personal resources, characteristics of the surrounding adults who explain to them the events they have experienced and school resources. These changes were detected despite the reduced proportion of words referring to mental states compared to the total number of words (about 7% of production), and the presence of a gradual increase in narrative abilities, in line with what has been documented by previous studies [19,25]. However, the comparison between the post- and the first lockdown period (T3 versus T2) also detects a decrease in psychological lexicon referable to cognitive states, which was not expected based on previous studies. This result merits further investigation. The analysis of the variations in the psychological lexicon in two matched groups (MG1 and MG2) of equal age and narrative skills, seems to confirm the peculiarity of the trend of terms referring to emotional states in the narratives written by children during the COVID-19 pandemic. Both matched groups performed the task in non-emergency health conditions, but at a comparable time interval to that used in the sample under study, of 5 and 12 months, respectively. In neither of the two matched groups there were significant changes in the use of terms referring to positive and negative emotional states or other emotional states.

The second study was aimed at investigating changes in socio/emotional and contextual properties in children’s drawing during the first and the third pandemic wave by asking children to create a drawing to “Represent yourself a year ago” and a drawing to “Represent yourself today”. Results showed that in the drawings referring to the third wave (“Represent yourself today”), when children went to school although following COVID-19 restrictions, contextual properties referring to pandemic tools (e.g., face masks) and settings (e.g., open spaces) in drawings were more often represented in comparison to the drawings representing the first lockdown (“Represent yourself a year ago”). Therefore, children reflect in their drawings their knowledge of functioning of the social relations in time of pandemic rules imposed by the legislative decrees, such as wearing a mask to protect against contagion, and at the same time the renewed possibility to leave home and return to social contexts. It is very interesting that the results linked to the reduction in closed spaces which positively correlates with the reduction in the use of dark colors and negatively correlates with the increasing use of light colors. In relation to this, the increasing use of light colors, reinforced by the reduction in the use of dark colors, might recall lighting, open physical and relational spaces compared to children’s drawings referred to the first lockdown. The latter trend was reinforced by the fact that there was a significant correlation between the reduction in the use of black and the reduction in the use of gray and that all the elements that refer to positive emotional experiences were interrelated (presence and closeness to other children and light colors).

Similarly to results in narratives, the results from drawings set in the direction to support that the first lockdown favored negative emotions in children [51]. Both narratives and drawings are configured as useful means of expression and communication of internal states for school-age children. From children’s narratives and drawing another important result emerged showing that children were able, after one year from the starting of the experience linked to COVID-19, to return to normal levels of emotional expression, recovering all that range of positive emotions that was expressed by them both in narrative writing and drawing.

Overall, the results of this research provide interesting practical implications and useful insights to foster the psychological care of children experiencing intense distress, such as a pandemic outbreak. A first consideration focuses on the increasing psychological lexicon in narratives coinciding with the return to a “pseudo-normal school”. Forced social distancing, adopted to contain the first spread of COVID-19, may jeopardise children’s emotional and behavioural development and mental health in the long term, much more than is currently understood [52]. This is in line with the study by Orben and colleagues [53] demonstrating that globally enforced physical distance may have long-term detrimental consequences on everyone’s emotional and mental health regardless of age, and that, on the other hand, the removal of sources of face-to-face social connections from everyday life has a particular effect on children, for whom peer interactions and social connectedness are critically important aspects of their development. The curricular activity of the primary school years is based precisely on the co-construction and active participation of children in group activities that make the classroom a true “community of discourse” [54]. The interruption of such practices and of a participatory classroom climate requires, first of all, teachers to reflect on ways to meet the needs of children in order to participate in routines of motivating character of social encounter and confrontation [29,55], which will remain primary even in critical conditions of school life.

In this perspective, teachers, school practitioners, and clinicians may use narratives and drawings as a means to explore variations in the emotional states and needs that a child, especially a young one, tends not to declare explicitly or on direct request. To this end, written narration and drawings are confirmed as practices that can combine inner reflection and access to one’s own mental states with the need for social-relational sharing [56] and that can be early used. Psychological lexicon and the analysis of drawings may be used as a channel to understand children’s emotions, expression, and regulation in individual or group sessions and, possibly, to favor the implementation of preventive interventions. The co-construction of invented stories can also be a useful tool for teachers to promote in children a circulation of shared emotional experiences in the classroom within a relational teacher-children scaffolding that act as a cognitive and emotional support.

It is important to underline some limitations of the study. In the first study, the sample size is small, as only one II grade class was taken into account. Furthermore, it was not possible to control any interfering variables related to having collected the stories produced by the children in the home context during the lockdown (T2), as opposed to the other phases of the study (T1 and T3) in which the narratives were produced by the children at school. Another limitation is having used the questionnaire [45] at the level of the class group in Study 1: this has prohibited an analysis of individual data and a possible in-depth survey on the relationship between what the parents reported and the emotional expression that emerged from the narratives produced by the children. Further, it is important to underline that the objective of the study was defined after the onset of the pandemic emergency, following qualitative observations on the sudden changes expressed by children in their productions. This aspect could have favored a non-representativeness of the sample involved in the present study.

Future research is needed to confirm the data of the present study, controlling further intervening variables, such as the family context, in larger and more representative samples; the results collected underline the need to make the use of structured but ecological procedures for listening to children’s emotions during everyday school life and family practices [57]. The analysis of the emotional contents, expressed through drawings and lexicon in written narratives, may thus represent an indirect, and therefore non-invasive, method for gathering individual and collective emotional and relational needs in children living under unpredictable and critical circumstances. Moreover, the effects of quarantines could extend beyond the period of enforced physical removal and, consequently, affect the population for years to come [55]. Children with psychological discomfort may manifest it through a wide range of symptoms, even in the months following the end of the emergency [58]. Thus, the analysis of emotional expressions through drawings and narratives can also provide an index of the long-term effects of social deprivation and reduced face-to-face interaction on children’s emotionality.

## Figures and Tables

**Table 1:** Means and standard deviations of the narrative skills measures obtained in the SSG at the three observation times; comparison with the data reported by Gamannossi & Pinto [25] in a sample of 80 children of grade II of a primary school.

	T 1 (November 2019) Mean (SD)	T 2 (April 2020) Mean (SD)	T 3 (October 2020) Mean (SD)	Gamannossi & Pinto [25]
Narrative length (*n*. of words)	98.33 (49.0)	133.71 (51.8)	109.19 (49.5)	*-*
Structure (0–5)	3.52 (0.8)	3.62 (0.8)	3.50 (0.9)	3.87 (1.01)
Cohesion (*n*. of connectives)	2.38 (1,9)	2.71 (1.1)	3.38 (1.9)	2.01 (0.82)
Coherence (*n*. of incoherencies)	0.33 (0.6)	0.24 (0.5)	0.23 (0.4)	0.94 (0.35)
Total score	6.23 (2.3) IC (95%) *: 5.17–7.30	6.57 (2.4) IC (95%) *: 5.46–7.67	7.09 (2.4)	6.82 (1.54)

* CI = Confidence interval.

**Table 2:** Means and standard deviations of the psychological lexicon measured in the SSG at the three observation times and the results of the analysis with Wilcoxon’s W-statistic.

Category	T1 (November 2019) Mean (SD)	T2 (April 2020) Mean (SD)	T3 (October 2020) Mean (SD)	W T2-T1 (*p*<)	W T3-T1 (*p*<)	W T3-T2 (*p*<)
Physiological state	1.10 (1.4)	0. 95 (0.1)	0.95 (1.1)	46 (ns)	76.5 (ns)	61 (ns)
Perceptual state	0.86 (0.9)	0.76 (0.9)	0.81 (1.2)	49 (ns)	77 (ns)	49.5 (ns)
Positive emotions state	1.76 (0.1)	0.95 (0.9)	1.52 (1.5)	**96 (0.01)**	81.5 (ns)	47 (ns)
Negative emotions state	0.33 (0.6)	1.29 (0.1)	0.62 (0.8)	**126 (0.005)**	28 (ns)	**93.5 (0.055)**
Willingness state	0.67 (1.1)	1.19 (1.0)	1.09 (1.7)	24 (ns)	46.5 (ns)	67 (ns)
Cognitive state	0.95 (1.4)	1.00 (1.1)	0.38 (0.6)	46.5 (ns)	44.5 (ns)	**85 (0.05)**
Moral state	0.38 (0.5)	0.62 (0.1)	0.43 (0.7)	36 (ns)	20 (ns)	46 (ns)
Socio-relational state	1.00 (1.4)	0.71 (1.1)	0.53 (0.9)	56 (ns)	49.5 (ns)	38 (ns)
Total	6.9 (3.2)	7.3 (3.3)	6.2 (3.3)	244.5 (.05)	96.5 (ns)	150 (ns)

Bold numbers means significant results.

**Table 3:** Narrative skills measures obtained in the groups MG1 and MG2 at the respective times of detection.

	MG1 T1 (January II) Mean (SD)	MG1 T2 (May II) Mean (SD)	MG2 T1 (May/June II) Mean (SD)	MG2 T2 (May/June III) Mean (SD)
*n*. of words	106 (42.9)	112 (28.9)	93.0 (31.7)	123 (54.8)
Structure (0–5)	3.10 (0.89)	3.14 (0.91)	3.0 (0.71)	3.48 (1.1)

**Table 4:** Frequency of the psychological lexicon used by MG1 MG2 in the respective times of detection and comparison for paired groups in the Wilcoxon test.

Category	MG1 T1 (January II) Mean (SD)	MG1 T2 (May II) Mean (SD)	W T2-T1 (*p*<)	MG2 T1 (May/June II) Mean (SD)	MG2 T3 (May/June II) Mean (SD)	W T3-T1 (*p*<)
Physiological state	0.62 (1.02)	0.81 (1.47)	19.5 (ns)	2 (1.58)	2.05 (1.6)	79.5 (ns)
Perceptual state	0.67 (0.86)	0.81 (1.47)	60.5 (ns)	0.76 (0.99)	1.2 (1.6)	49 (ns)
Positive emotions state	1.19 (0.98)	1.24 (1.22)	43 (ns)	1.38 (1.63)	0.95 (1.32)	106 (ns)
Negative emotions state	0.57 (0.67)	0.43 (0.98)	55.5 (ns)	0.24 (0.54)	0.52 (75)	22 (ns)
Willingness state	0.43 (0.68)	0.52 (0.87)	24.5 (ns)	0.76 (0.94)	1 (1.38)	41.5 (ns)
Cognitive state	0.33 (0.48)	0.52 (0.87)	25 (ns)	0.3 (0.57)	0.67 (0.80)	26.5 (ns)
Moral state	0.33 (0.65)	0.57 (0.87)	20 (ns)	0.81 (1.03)	0.19 (0.51)	47 (ns)
Socio-relational state	0.38 (0.49)	0.52 (0.51)	24 (ns)	0.95 (1.4)	0.19 (0.40)	64.5 (ns)
Total	4.10 (1.07)	5.43 (4.03)	138 (ns)	7.24 (2.6)	6.77 (4.2)	114 (ns)

**Table 5:** Means and Standard Deviations of drawings’ analysis.

Drawing Codifying System	Drawing Tasks		W (*p*<)
	Represent Yourself a Year Ago M (SD)	Represent Yourself Today M (SD)	
Social/emotional properties			
Proximity with other figure/s	2.15 (1.76)	2.08 (1.6)	z = −0.51, ns
Presence of figure/s	2.10 (1.68)	2.06 (1.49)	z = −0.11, ns
Use of different colors			
***Gray***	1.97 (1.12)	1.73 (1.02)	z = −1.88, *p* = 0.06
***Black***	1.79 (1.01)	1.52 (0.71)	z = −2.49, *p* < 0.05
***Yellow***	2.42 (1.48)	2.44 (1.55)	z = −0.4, ns
***Green***	2.72 (1.77)	2.83 (1.74)	z = −0.69, ns
**Derived index of *Dark Color***	1.88 (0.9)	1.62 (0.73)	z = −2.45, *p* < 0.05
**Derived index of *Light Color***	2.57 (1.41)	2.63 (1.39)	z = −0.55, ns
Contextual properties			
**Presence of physical close environment**	4.31 (2.31)	3.7 (2.45)	z = −2.16, *p* < 0.05
**Presence of pandemic restriction elements**	1.24 (1.05)	2.84 (2.42)	z = −5.93, *p* < 0.001

**Table 6:** Correlations between all indexes.

	1	2	3	4	5	6
1. Delta Proximity with other figure/s						
2. Delta Presence of figure/s	0.57 **					
3. Delta Dark Colors	−0.10	−0.09				
4. Delta Light Colors	0.31 **	0.21	−0.34 **			
5. Delta Presence of physical close environment	−0.16	−0.08	0.26 *	−0.43 **		
6. Delta Presence of pandemic restriction elements	−0.03	0.12	−0.07	0.04	−0.01	

Legend: ** *p* < 0.01; * *p* < 0.05.

## Data Availability

The data presented in this study are available on request from the corresponding author.

## Appendix A

**Table A1:** Description of the coding system for mental state talk (adapted from Bretherton and Beeghly, [59]).

1. Category	2. Description	3. Examples
4. Perceptual and physiological states	5. Terms referred to perceptual and physiological states that might influence behavior	6. Being hungry, feeling hot/cold
7. Emotional state	8. Terms referred to feelings and emotions (positive and negative)	9. Happy, sad, angry
10. Willingness state	11. Terms referred to what goals and aims	12. Willing, hoping, looking for
13. Cognitive state	14. Terms referred to thoughts and cognitive functioning	15. Knowing, understanding, forgetting
16. Moral and sociorelational state	17. Terms referred to the moral dimensions and the relationships between characters	18. Joking, helping, becoming friends

## Appendix B. Examples of Narratives by Children Structure: Respectively, of Level 2, Level 3, Level 4

**Example** **A1** 
*The house within the house*

*Two children had built a house. It was a very special house though! The walls were made from chairs, the roof was made from blankets, the wardrobe from a box, the floor from sofa cushions. Inside it had all the comforts: a light, a water bottle, biscuits and even a bookcase!!!*

**Example** **A2** 
*My invented story*

*Once upon a time there was a dog who lived on a big beach. The dog’s name was Spike and he was always sad. One day he heard music and he followed, opened the door of a hut and Cesar good fairy the fairy. The fairy adopted him and they were always happy together and Spike discovered that one has to be many to be happy.*

**Example** **A3** 
*Martina the animal curator*

*Once upon a time Martina was a friend of animals: she was a curator and one day she found a small brown dachshund dog with white ears wounded on one cheek abandoned named Lio. So Martina took her to the animal shelter and said: ‘This will be his home until you are adopted’. On the second day Lio tried to run away but his owner Martina prevented him because he still needed a lot of care. On Wednesday morning she brought a white cat with black stripes and a scratch on one paw called Ozzi. The two were put together in the same cage but they started to fight and so Martina split them up and the cat with a meow meows dog-cat war, because we all know that dogs and cats don’t get along. The war lasted for 10 days but for the leaders Lio and Ozzi it lasted 16 days and in the end who won? Nobody because Martina stopped them and so dog and cat, still somewhat enemies, made peace and so did the tribe. She explained to them that they had to start collaborating to make a good group, or a big family where they could have fun even if different.*
